# A Brief Focus on SARS-CoV-2 Genomic Evolution and Vaccines

**DOI:** 10.3390/pathogens12101253

**Published:** 2023-10-18

**Authors:** Annamaria Pratelli, Canio Buonavoglia

**Affiliations:** Department of Veterinary Medicine, University of Bari, Sp Casamassima Km3, 70010 Valenzano (Ba), Italy; canio.buonavoglia@uniba.it

Severe acute respiratory syndrome-coronavirus type 2 (SARS-CoV-2) emerged in a live animal market in the Hubei Province of Wuhan in China in late 2019 and was declared a pandemic by the World Health Organization (WHO) on 11 March 2020 [1].

Although the definitive origin of the virus remains uncertain, at least two major hypotheses prevail: (i) the accidental escape from a laboratory of a bat with coronavirus with subsequent spread among humans; (ii) a natural origin with the involvement of different animal species in close contact with each other, followed by human-to-human transmission [2,3]. Since some species of bats are recognized as the natural host for a wide range of CoVs, they represent the ideal “mixing vessels” for recombination events among strains of different origins, favoring the emergence of new viruses able to cross species barriers and to cause severe diseases in human [4]. The development of safe and effective vaccines was the most effective tool to tackle SARS-CoV-2 infection and to prevent the development of severe illness. Although vaccine development is generally a slow process that can take many years to complete, the availability of modern biomolecular technologies, and the genomic sequences of viruses in open sources, have favored the rapid (in mere months) development of a wide range of different vaccines [5]. Several candidate vaccines prepared using existing expertise in vaccinology were tested worldwide, with the best results to date obtained using mRNA technology, a novel lipid nanoparticle encapsulated mRNA encoding for the SARS-CoV-2 spike protein, and from the viral vector-based vaccines expressing the SARS-CoV-2 spike protein [2]. The most common viral vector-based vaccines use a chimpanzee adenovirus (AdVs) to avoid pre-existing immunity to some human AdVs, which could compromise their effectiveness [4]

Both vaccine platforms were shown to be immunogenic and to induce strong humoral and cell-mediated immune responses. Two doses of mRNA vaccines stimulate strong T-cell responses in more than 92% of the inoculated patients and show 100% seroconversion two weeks after the booster dose. The viral vector-based vaccines elicit both T-cell and IgG-antibody responses on day 14 and by day 28, respectively, which are improved with the administration of a booster [4].

Despite the strong scientific progress in the development of effective vaccines against SARS-CoV-2 and the excellent results achieved, several questions remain regarding the acquired immunity after infection but also after vaccination: (i) how long does vaccine protection last at both the humoral and cellular level? (ii) To what extent does the post-vaccinal cellular immune response persist? (iii) What is the degree of protection against variants?

The intriguing features of CoVs are their extremely large genome, their nested set of subgenomic mRNAs, their discontinuous transcription mechanism, and the high error frequencies of RNA polymerases, that are predicted to favor the accumulation of several base substitutions per round of replication and ensure the proliferation of new strains (variants) with probable selective advantages over parental genomes [6,7,8]. The extremely variable nature of CoVs led to the rapid and continuous onset of mutations (which can be neutral, beneficial or harmful) and recombinations. This potential for genetic evolution guarantees the proliferation of new strains, which could have selective advantages over the parental genomes and could allow for the overcoming of species barriers [9]. 

While switching the host and adapting to a new one, the receptor-binding domain of the S protein undergoes specific mutations, improving binding to the angiotensin converting enzyme 2 (ACE2) cellular receptor, and enhancing virus entry and virus replication in the host cell [10]. A recent study identified sixteen mutations in different domains of the S protein: thirteen mutations were found in the S1 subunit, and four in the receptor-binding domain (RBD), the main site responsible for ACE2-binding to which the majority of human antibodies are targeted [11]. It can therefore be hypothesized that SARS-CoV-2 infectivity and its ability to escape the immune response are also favored by the high rate of mutation events that occur in this domain, affecting antibodies’ binding [10,12].

Furthermore, some mutations can have an impact on both infectivity and replication, and the emerged strains could affect the effectiveness of current vaccines. From the perspective of successfully controlling the spread of SARS-CoV-2 with vaccines, maximum attention is thus paid to monitoring the genetic evolution of the virus and to the biological impact of the emerging variants [10,13]. 

On the contrary, S2 subunits are relatively more conserved, even if they contain the fusion peptide (FP) and the heptad repeat (HR1, HR2) regions. Among these, the FP region participates in the host cell infection thanks to an extended bipartite fusion platform, and, consequently, it could also be a potential target for the design of new vaccines [13,14].

The history of animal CoVs has demonstrated that genetic changes can give rise to mutations and/or (more dangerously) to recombination or to gain/loss mechanisms in accessory genes [15]. These drastic events could generate new viruses with modified virulence that are able to adapt to different hosts, avoiding the immune response [16,17]. Recently, in addition to the emergence of three human epidemics/pandemics caused by CoVs, a closely related but distinct canine coronavirus (CCoV) with a unique deletion in the nucleocapsid protein was identified in the nasopharyngeal swabs of children with pneumonia in Malaysia [18]. Furthermore, in Haitian paediatric patients, a CoV that likely jumped from pigs to people was reported [19]. Although animal-to-human transmission has already occurred in the past, in the last decades its occurrence appears to have increased and involves viruses with zoonotic potential. All these events, in addition to the onset of variants, must represent a warning regarding the recombination events which are a very dangerous characteristic of CoVs [15].

The use of vaccines allows for controlling the viral spread and containing the pandemic, but recent events have dramatically highlighted how little we know about CoVs and, in particular, their ability to jump between species (i.e., animal-to-human) and how thin the human–animal barrier is. Our acquired experience in the patho-biology of animal CoVs, today more than ever, should be applied to enhance our knowledge of SARS-CoV-2 and, above all, applied in the development of effective vaccines.
